# Progress in Fevers

**Published:** 1902-08-30

**Authors:** 


*(To be continued.)*

### Progress in Fevers.

**Plague.**—The Report of the Indian Plague Commission, of which Professor S. R. Fraser, of Edinburgh was President, and which included Professor Wright, of Netley, consists of several large foolscap volumes. Of these Vol. V., the Final Report, is the most important. It contains seven chapters and five appendices.[1] Chapter I. contains chiefly formal matter. Chapter IV. was published in February 1900. It deals with Haffkine's anti-plague inoculation.[2] Chapter II. gives an account of plague in India from 1896. The disease, practically unknown in the country for 60 years, was introduced into Bombay, obscurely, but probably by sea, in July 1896, and has chiefly affected the presidency of Bombay, showing a yearly decline and increase from 1896 to 1899, the period chiefly covered by the Report. Disinfection, isolation, removal to hospitals and camps, were practised as vigorously as possible. So extensive was the exodus of the inhabitants that in Bombay City a normal population of 846,000 fell to 450,000 in February 1897. In 1898 segregation of contacts was partially practised. During March 5,000 deaths from plague occurred, whilst only 200 deaths were reported in June, and a similar decline in June was noticed next year. Altogether 41,000 deaths from plague were reported, and probably 30,000 more actually occurred in Bombay between January 1897 and June 1899. Plague raged to a less extent in other parts also—in Mysore, Bengal, Madras Presidency and the Punjab and North-Western Provinces. Chapter III. deals with the characters of plague and the agents by which infection is spread. An appendix on the clinical features and treatment is supplied by the President, who, considering the serumtherapy of plague, remarks: "On the reasonable supposition that in bubonic cases the plague bacilli are for a time concentrated in the primary buboes, it is advisable to inject the serum into the buboes or the tissues immediately surrounding them, and it is probable that the best results will be obtained by this plan of administration, which, however, does not appear as yet to have been employed in practice." In an "average case" the incubation period was found to be well within five days. Pneumonic plague is classed as "highly infectious," but the infectivity of plague by personal contract under conditions of good ventilation and hygiene, such as obtain in a well-managed hospital, is considered small. Summing up concerning the infectivity of plague, the commissioners decide that the bubonic form is only dangerous through the excretions and in the last stages, that the primary pneumonic form is highly infective, that houses inhabited by plague patients or by plague-stricken rats and soiled fomites are infective, and that living in an infected house is far more dangerous than contact with a plague patient. Plague is a "disease of locality." The commissioners are guarded in their views regarding rats. They think the chief importance to be attached to their agency is the carrying of the disease to a hitherto unaffected place—"in many instances . . . they scatter plague broadcast over an uninfected place." Evidence was given showing that a month or two may elapse between the importation of infection and the outbreak of the disease. During this interval the commissioners think infection persists in soil or clothes rather than among rats. Professor Fraser thinks plague "cannot justly be regarded as a filth disease in the ordinary acceptation of the term," but the moisture and warmth required by its bacillus are found in unventilated rooms. Serum therapeutics are reviewed in Chapter V. The commissioners found Yersin's serum innocuous to guinea-pigs, they therefore used it for some hospital patients, but without striking success. Case mortality showed a diminution of not more than 4 or 5 per cent. Lustig's serum, which was found to accelerate death in guinea-pigs, was not employed. Several independent observers, however, reported diminished case mortality in patients treated with serum. The German and Russian plague commissions both found the serum harmless but useless. The Indian commissioners' own experiments were on a small scale and unsatisfactory. In Chapter VI are detailed the methods adopted for obtaining information of plague cases, such as notification, house-to-house visitation, corpse inspection, etc., and also the measures employed to check the spread of the disease, such as removal to hospitals, segregation of contacts, evacuation and disinfection—chemical and by means of admitting sunlight and air. The President regards free ventilation as of great importance, but the majority of the commissioners consider diffused daylight has no efficacy as a disinfectant. Most of these measures met with more or less opposition among the people. The commissioners hold that "if every case of plague could be isolated at once, and if the personal effects, clothes, and house of a patient were disinfected, and, further, if all plague-infected rats

DAMAGE

were removed and burnt, and the houses in which these had been found were disinfected, and finally, if the healthy persons who are exposed to infection would be inoculated, the ravages of plague would be minimised." In spite of all that has been done, however, they reach the sorrowful conclusion that "there are no means of stamping out the present epidemic of plague." Chapter VII. is devoted to important recommendations and suggestions regarding the organisation of the Sanitary Department in India, which at present is defective in important particulars in its equipment for grappling with the disease. As pointed out elsewhere,[3] outside the larger towns there appears to be practically no sanitary administration at all, and medicine and sanitary science are not represented on the Council of the Viceroy of India, nor on that of the Lieutenant-Governor of Burmah, although engineering, law, commerce, etc., are directly represented. C. H. Hunter,[4] in reviewing the report of the commission, emphasises some additional points. He shows that although the rôle played by rats in the spread of the disease is abundantly supported by the evidence laid before the commissioners, the association between rat plague and human plague has not always been well marked. Epidemics at Oporto, Alexandria, Jeddah, and other places have shown no close association between the two, although sometimes high plague mortality continued among the rats *after* the human epidemic had ceased. During severe epidemics also in India in recent years, as at Poona and Hubli, few or no dead rats were observed. Infection of rats through the alimentary canal the commissioners think is uncommon—ten rats persistently fed on plague infected food remained healthy. Infection through the mucous membrane of the nose is considered most likely. Again emphasis is laid on the fact that, in the view of the President, plague is undoubtedly spread and rendered more virulent by insanitary conditions, especially those prevailing *inside* the houses, such as deficient light and ventilation, overcrowding, dampness, a polluted atmosphere, and, to a less extent, want of cleanliness. No connection can be shown to exist between outbreaks of plague and atmospheric temperature and moisture, or rainfall, such as Pettenkofer has demonstrated as existing in the case of cholera. Pettenkofer first showed that the biological activity of a soil is best measured by the amount of carbonic acid contained in the ground air, and Lewis and Cunningham have shown that the time of maximum cholera prevalence coincides with the time of maximum soil ventilation, or with the time of minimum amount of carbonic acid in the ground air at a depth of 3 feet or 6 feet. When the atmospheric air is colder and therefore heavier than the ground air at a depth of 5 or 6 feet, the latter will escape into the atmosphere at places of least resistance, *e.g.* the earth floors of the native Indian houses at night in the cold season. Such conditions exist during the months of maximum plague incidence in Bombay. Dr. Marsh found that agar cultures of plague bacilli flourished virulently in soil well impregnated with carbonic acid gas, and also in an artificial atmosphere charged with 3 per cent. of the gas. The theory of the acclimatisation of the plague bacillus advanced by Major Anderson and supported by reference to some bacteriological work of Danyz is rejected in the report. The medical congress recently assembled at Hobart[5] resolved that the name Pestis Minor should not be applied to non-plague cases. The term, if used at all, should be restricted to mild cases of plague, otherwise there is danger, as shown recently at Brisbane, of the public making light of mild cases of plague. In January last an outbreak of plague of some severity occurred in Bagdad.[6] Infection is believed to have lingered on from some mild cases of glandular fever, which probably were true cases of mild plague. In the recent epidemic at Sydney[7] a plague-stricken rat was several times discovered in a new quarter of the city before a human case appeared. Some interesting details are forthcoming from Marseilles regarding the precautions taken at that port to keep plague at bay.[8] For many years the island of Frioul, a few miles out, has been used as a disinfecting and quarantine station. By means of an ingenious system of automatically working machinery all cargo, baggage, clothing, &c., is thoroughly disinfected and treated with superheated steam in stoves. Passengers and crew are all in turn conducted to a cabin divided into three compartments. In the first all clothing is removed and handed out for disinfection, in the second a bath is taken, while in the third the now disinfected clothing is resumed. Twenty such compartments are provided and 250 passengers have been thus disinfected in a single morning. An outbreak of plague last year on the ship *Sénégal*, which was taking a number of French *savants* and scientists for a cruise in the Mediterranean, occurring 27 days after the ship had left the plague-infected port of Alexandria, and after thorough disinfection at Marseilles, directed attention to the rats on board, which were found to be suffering from plague. Since this incident careful attention has been given to the destruction of rats on board ships coming from infected ports as well as to the disinfection of ship, occupants, and cargo. Regarding serum treatment,[9] Yersin found that the samples of serum he used with success in China, though weak, retained their properties for some time after preparation. He found the earlier the treatment the better the result. Other workers, as Symmers, Krumbein, and others, speak of the great difficulty and length of time involved in procuring an efficient serum. Stewart found that he obtained a less effective serum when he inoculated a horse with a nucleo-proteid extract of the dead microbes than when he used the dead bacilli themselves. Living bacilli were still more efficacious. Yersin's serum reduced mortality in the Oporto outbreak of 1899. Subcutaneous injections (40 c. cm.) and intravenous injections (20 c. cm.) combined were found best. Roux-Yersin's serum was also useful in the Buenos Ayres epidemic, especially if large doses were given early. Brownlee found subcutaneous injections of Yersin's serum of little value at Glasgow in 1900. He thinks the lymphatic system acts as a biological filter on the serum, and that the antitoxins are retained by the glands draining the area of injection. He recommends large initial doses—60 c. cm.—given intravenously.

[1] Brit. Med. Jour., May 3, 10, 17, and 24. [2] Ibid., 1900, Vol. I., p. 455. [3] Ibid., May 24, p. 1288. [4] Edin. Med. Jour., Feb [5] Australasian Med. Gaz., May 20. [6] Lancet, Feb. 22. [7] Brit. Med Jour., May 31, p. 1377. [8] Lancet, May 24 and 31. [9] Brit. Med Jour., June 14, p. 1492.

(*To be continued.*)

